# Functional Electrical Stimulation Plus Visual Feedback Balance Training for Standing Balance Performance Among Individuals With Incomplete Spinal Cord Injury: A Case Series

**DOI:** 10.3389/fneur.2020.00680

**Published:** 2020-07-23

**Authors:** David J. Houston, Jae W. Lee, Janelle Unger, Kei Masani, Kristin E. Musselman

**Affiliations:** ^1^KITE Toronto Rehab-University Health Network, Toronto, ON, Canada; ^2^Faulty of Medicine, Rehabilitation Sciences Institute, University of Toronto, Toronto, ON, Canada; ^3^Faculty of Engineering, Institute of Biomaterials and Biomedical Engineering, University of Toronto, Toronto, ON, Canada; ^4^Department of Physical Therapy, Faculty of Medicine, University of Toronto, Toronto, ON, Canada

**Keywords:** visual feedback, balance training, functional electrical stimulation, spinal cord injury, neurorehabilitation

## Abstract

Individuals with an incomplete spinal cord injury (iSCI) are highly susceptible to falls during walking or standing. Our objective was to evaluate a therapeutic tool for standing balance that combined functional electrical stimulation, applied bilaterally to the plantarflexors and dorsiflexors, with visual feedback balance training (FES+VFBT). Five adults with iSCI completed 12 FES+VFBT sessions over 4 weeks. During the training sessions, participants completed each of the four balance exercises twice. Visual feedback of the center-of-pressure (COP) location was provided as participants completed the balance exercises and received FES to assist with performance of the exercises. A closed-loop FES system was used in which the COP was continually monitored and the level of electrical current administered was automatically adjusted. Balance abilities were assessed pre- and post- training using clinical balance scales (i.e., Berg Balance Scale, Mini-Balance Evaluation Systems Test, and Activities-specific Balance Confidence Scale) and biomechanical assessments (i.e., postural sway measures and limits of stability test during standing). User acceptability was explored through semi-structured interviews. Improvements were seen for four of the five participants on at least one of the clinical scales following completion of the training intervention. All participants showed greater maximal COP excursion area during the limits of stability test after the training intervention, whereas only one participant demonstrated a reduction in postural sway. Specific components of FES+VFBT, including the ability to safely practice challenging balance exercises, were deemed important by the participants. These results suggest that FES+VFBT has potential as an intervention for standing balance after iSCI.

## Introduction

Sustaining a spinal cord injury (SCI) is a life-changing event that challenges the individual's level of independence, mobility, and overall quality of life. Damage to the spinal cord produces sensorimotor changes below the level of injury that can occur as a result of traumatic (e.g., motor vehicle accident or fall) or non-traumatic (e.g., tumors or infections) causes.

Individuals with a motor incomplete SCI (iSCI) retain some residual motor functioning below their level of injury. Indeed, the majority of these individuals regain the ability to walk in the community at 1-year post-injury (1). Their sensorimotor impairments, however, reduce their ability to modify their movements relative to task demands, thereby affecting their balance control and increasing their risk for falling (2, 3).

Falls are of significant concern among individuals with iSCI due to the likelihood of injury or hospitalization (4). Each year, 78% of ambulatory individuals with iSCI sustain at least one fall (5), often during periods of standing or walking within their own homes (6, 7). The occurrence of a fall, regardless of injury, can produce changes in behavior that stem from a learned fear of falling and are intended to restrict an individual's level of mobility (8). This can severely limit an individual's ability to engage in meaningful activity and participate in their community (6, 9).

Under conditions of normal quiet standing, upright balance is maintained through small postural rotations around the ankle joint which are dependent on the amount of ankle stiffness present within the joint (10). Different sensory inputs (i.e., vision, somatosensory, vestibular) are integrated to modulate the neural inputs to the plantarflexor muscles and adjust the amount of ankle stiffness (10). Lemay et al. (11) showed that individuals with SCI were less stable during stance than able-bodied individuals and exhibited greater dependency on visual inputs to maintain control of their balance [i.e., maintaining their center of pressure (COP) within their base of support] during standing. This increased reliance on visual inputs has provided an opportunity to incorporate visual feedback into the rehabilitative process for balance control. Visual feedback balance training (VFBT), which involves the visual representation of the COP locations during balance exercises, has been shown to be an effective means to improve postural control of balance among individuals with iSCI (12, 13).

As the ankle muscles play an important role in maintaining standing balance, interventions that induce the activation of the weakened ankle muscles may be a beneficial complement to balance training within the motor iSCI population. Functional electrical stimulation (FES), which applies an electrical current to the peripheral nervous system to produce muscle contractions within the context of functional task performance (14), can lead to increased strength of corticospinal connections (15) and increased motor unit recruitment in the muscles of individuals with upper motor neuron damage (16). While FES has been used in the rehabilitation of upper extremities (17) and gait (18) after motor iSCI, it has only recently been considered as a complementary intervention to standing balance training for this population.

Our team developed a closed-loop FES system that targets the ankle musculature during standing (19–21). This system continuously monitors the position and velocity of the body and automatically adjusts the level of electrical current administered to the plantarflexors and dorsiflexors bilaterally. The closed-loop FES system effectively mimics the physiological control system used by able-bodied individuals to maintain standing balance control (22, 23). Recently, a novel therapeutic tool integrating FES and VFBT (FES+VFBT) has been developed and validated among young, able-bodied individuals (24, 25). However, the use of this system has not been evaluated within a neurological population.

Here we evaluated the therapeutic potential of the FES+VFBT system for standing balance control in five individuals with chronic, motor iSCI. We hypothesized that following the FES+VFBT intervention, participants would show improved balance control, as demonstrated by improved performance on clinical balance scales [i.e., Berg Balance Scale (BBS), mini-Balance Evaluation Systems Test (mini-BESTest), and Activities-specific Balance Confidence (ABC) Scale] and biomechanical assessments (i.e., postural sway measures and limits of stability test during standing). We also hypothesized that these improvements would be retained at follow-up assessments completed 4 and 8 weeks post-FES+VFBT intervention. In addition, user acceptability of the FES+VFBT system was explored through semi-structured interviews.

## Methods

All study activities occurred at the Lyndhurst Center, Toronto Rehab-University Health Network. Research ethics approval was obtained from the University of Toronto and the University Health Network. A case series following a single-subject experimental design with both quantitative and qualitative evaluations was used to evaluate FES+VFBT as an intervention for standing balance. Using a single-subject experimental design allowed for each participant to serve as their own control for pre-post assessments (26). It is an appropriate study design when evaluating the feasibility of a new intervention or technology, especially in a condition with considerable heterogeneity, like iSCI (27, 28).

### Participants

Individuals with an iSCI were recruited via flyers posted at the Lyndhurst Center and 15 potential participants were assessed for eligibility upon obtaining written consent.

Following the screening process, five adults (1 male, 4 females) with motor iSCI [i.e., American Spinal Injury Association Impairment Scale (AIS) rating of C or D] were enrolled (see Table 1). All participants were at least 12 months removed from injury or the onset of neurological symptoms in the case of non-traumatic iSCI, were capable of unassisted standing for 60 s, and had a BBS score <46. Four individuals were deemed ineligible during the screening process and six individuals declined to participate in the study due to their inability to commit for the duration of the study or a lack of transportation. Before beginning FES+VFBT, participants completed baseline assessments over a period of 4 weeks. They then completed 12 FES+VFBT sessions over 4 weeks. Follow-up assessments were completed at 4 and 8 weeks post-FES+VFBT.

**Table 1:** FES+VFBT participant demographics.

**Participant**	**Age range**	**Cause of injury**	**Level of injury**	**AIS classification**	**Time post-injury (months)**
1	65–69	Staph Infection	T6	C	97
2	65–69	Virus	C5	C	52
3	60–64	Fall	C1	D	22
4	60–64	Surgery	C3	D	32
5	55–59	Surgery	T10	C	31

*C, cervical; T, thoracic; AIS, ASIA Impairment Scale*.

### Clinical Assessment

**Figure 1:**
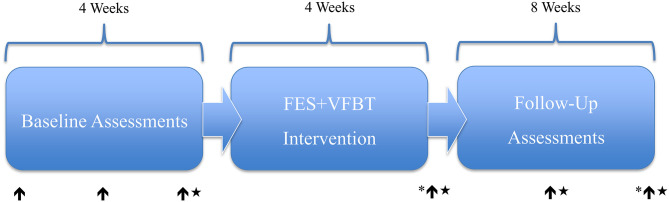
FES+ VFBT experimental timeline and assessment points. ↑ = Clinical Assessment. ★ = Biomechanical Assessment. * = Semi-Structured Interview.

A physical therapist, blind to the study aim and intervention, administered the BBS, Mini-BESTest, and ABC scale. Each scale has been shown to be a valid and reliable measure for individuals with chronic motor iSCI (3, 29, 30). The BBS is used to assess balance performance during 14 sitting or standing tasks. Each test item is scored by the examiner on an ordinal scale of 0 to 4 based on the time or distance requirements and the need for assistance or supervision from the examiner (31). The highest attainable score is 56 and the minimal detectable change (MDC) is 4.4 points (32). The mini-BESTest uses a 0 to 2 ordinal scale to assess balance on 14 items divided into four categories: anticipatory, reactive postural control, sensory orientation, and dynamic gait (33). The highest attainable score is 28 and the MDC for the iSCI population is 4.67 points (34). The ABC Scale requires participants to rate their perceived confidence in their ability to perform 16 different standing and walking activities (35). For each activity, a value between 0% (no confidence) to 100% (completely confident) is assigned to denote how confident they were that they could complete the task while maintaining their balance. The total ABC Scale score is the average score of the 16 different activities and a MDC of 14.87% has been reported for individuals with chronic motor iSCI (30). Clinical balance assessments were administered prior to beginning the balance training intervention (three times over 4 weeks), after completion of training, and 4 and 8 weeks after the balance training intervention was completed (see Figure 1).

An average of the three baseline measurements was determined for each participant. Standard deviation (SD) was calculated to capture the variability in baseline performance. Change in performance was reported relative to the average baseline value and evaluated using the two-standard deviation band method (26) for each participant. Each band includes values two SD above and below the mean baseline performance for the participant and is used to determine where subsequent values lie in comparison to the initial band (i.e., within 2SD from the mean) (36). Values falling outside two SD were interpreted as a true change in performance.

### Biomechanical Assessment

The biomechanical assessment was completed once prior to beginning FES+VFBT, once after the completion of the training, and at 4 and 8 weeks following the completion of the intervention (see Figure 1). Two biomechanical tests were completed with the participants standing while secured in an overhead harness for safety. First, the static balance test was completed by measuring postural sway during quiet standing with eyes opened, followed by the dynamic balance test where the limits of stability were evaluated. For both tests, participants' feet were placed on two adjacent force plates, with one foot on each force plate (AccuSway-Dual, Advanced Mechanical Technology Inc., Watertown, USA), as they stood with both arms crossed across their chests. All force plate data were sampled at 2,000 Hz and a 4th order low-pass Butterworth filter (4 HZ for quiet standing; 10 Hz for limits of stability) was used. All off-line calculations were performed using a custom-written code in a computing language (MATLAB R2019, The MathWorks Inc., Natick, MA). We also collected kinematic data using motion capture data, which was not used for this study.

For the static balance test, participants were instructed to stand still for 60 s while focusing on a circle located at eye level on the computer monitor. Two quiet standing trials were performed with a short rest in between trials. To characterize postural sway, COP velocity and the root-mean-square (RMS) of the COP displacement were calculated in both the anterior-posterior (AP) and medial-lateral (ML) directions. These postural sway measures are valid and reliable for the iSCI population (37). Each quiet standing trial was divided into two 30 s windows. Mean COP velocity and COP RMS displacement were determined in each window for both AP and ML. Hence, four values (2 trials × 2 windows per trial) for each of AP COP velocity, ML COP velocity, AP COP RMS, and ML COP RMS were obtained. The mean and SD of each measure were determined for each assessment time point. Changes in performance on the postural sway measures were reported relative to the average baseline value and evaluated using the two-standard deviation band method.

For the dynamic balance test, participants were asked to shift their COP in one of eight directions, offset by 45 degrees, as indicated by a target displayed on the computer monitor. As the individual leaned in each of the directions, a red dot visually representing their COP (calculated from the force plates and previously determined using the quiet standing trials to set the origin) moved accordingly to provide visual feedback. Participants were instructed to shift their COP as far as possible and hold their maximal endpoint for 2–3 s before returning back to their initial standing position. The dynamic balance test was performed twice with a short rest between trials. To characterize performance on the dynamic balance test, maximal COP excursion was reported relative to the ankle joint using the mean of the two collected trials. Maximal COP excursion was calculated using peak COP displacement recorded from the force plate data and relative to the position of the ankle joint. In addition, total sway area during the dynamic balance test was calculated using the sum of the area of eight triangles (Equation 1) corresponding to the maximal COP endpoint of each direction.

(1) $$\begin{array}{l} \text{Area}=\overset{8}{\underset{i=1}{\sum }}\left[\frac{\sqrt{2}}{4}\left(l_{1}{\;}^{*}\;l_{2}\right)\right] \end{array}$$

Where *l*_1_ and *l*_2_ are the maximal COP excursion in two successive directions.

A change in performance was expressed as a percentage relative to baseline performance (Equation 2) for each assessment time point.

(2) $$\begin{array}{l} \text{Change in Performance}=\left(\frac{y-x}{x}\right)*100 \end{array}$$

Where *x* = baseline value and *y* = value at post-training, or 4 or 8 weeks post-training.

### FES+VFBT

Participants completed three 1 h training sessions per week for 4 weeks, resulting in a total of 12 training sessions. Each training session consisted of 15 min to identify the motor thresholds and maximum tolerable stimulation levels for the ankle dorsiflexors and plantarflexors, 5 min to don/doff the safety harness, 5 min to calibrate the VFBT exercises, 20 min to complete the VFBT exercises, and 10–15 min to take rest breaks between exercises as needed. Tests of quiet standing and limits of stability, as described above, were completed prior to each training session to determine the average COP location during the natural standing posture and to identify the range of COP movements in order to calibrate the VFBT exercises for each individual.

**Figure 2:**
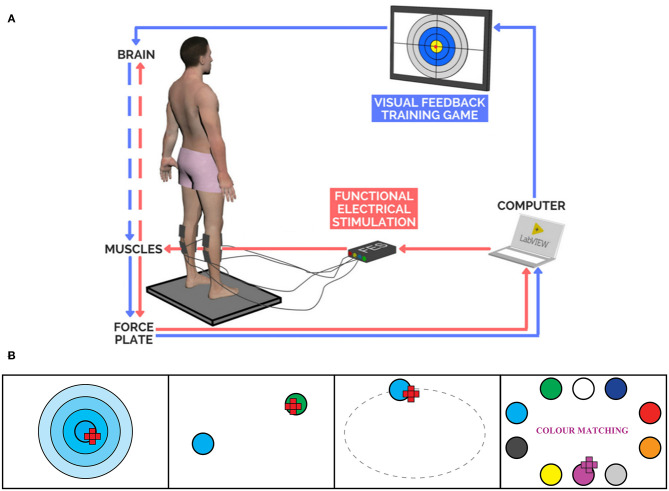
**(A)** FES+VFBT System (25). **(B)** Interfaces of VFBT Exercises: Bullseye, Hunting, Ellipse, and Color Matching, respectively from left to right.

The FES+VFBT system consists of a VFBT component and a FES controller (Figure 2A). During the VFBT exercises, visual feedback was provided regarding COP location (i.e., visually represented as a red dot on the computer monitor). Four different training exercises were performed as part of the balance program (Figure 2B). Each exercise was performed for 100 s and was completed twice per training session.

1. **Bullseye:** A large target was presented in the center of the screen. Participants were instructed to stand as still as possible to try to maintain their COP within the center of the “bullseye.”
2. **Hunting:** Participants were required to shift their COP toward a randomly presented target located within one of four quadrants on the computer monitor. The target would turn green once the participant managed to shift their COP inside the presented target. A new target in a different location would appear after 15 s had passed or if the individual was able to accumulate five total seconds of their COP within the target. Participants were then told to repeat the task with the newly presented target. The number of targets successfully “cleared” was presented in the top right corner of the computer monitor.
3. **Ellipse:** Participants were required to track a target as it moved around an ellipse on the computer monitor in either a clockwise or counter-clockwise manner. The target traveled at a constant speed around the ellipse. Individuals were instructed to shift their COP and track the target around the ellipse. When the COP was within the target, the target turned green. The percentage of the ellipse traveled was displayed on the computer monitor.
4. **Color Matching:** Participants were presented with color-coded targets located around the edges of the computer monitor. Large text that read “Color Matching” was located in the middle of the computer monitor and would turn the color of the desired target. The participant was instructed to locate the target matching the color of the text and shift their COP toward that target. Once the COP was within the target, the target would turn green. Participants were instructed to remain within the target until a new color was presented. Colors changed after 15 s had elapsed or if the COP was maintained within the target for a total of 5 s. The number of colors successfully “matched” was displayed on the computer monitor.

The FES controller used in the present study was an extension of the closed-loop FES control system developed in previous studies (19–21) and included gravity compensation and directional biasing (24, 25). A proportional-and-derivative controller served to regulate ankle torque to assist the participant with moving their COP to the onscreen target. In addition, a gravity compensation component acted to regulate ankle torque to support the subject and help them maintain their current lean in the AP direction. Since the proportional-and-derivative controller in our previous studies (19–21) only considered movement in the AP direction, the current system incorporated a directional biasing component designed to modify the stimulation intensity in a similar behavior to natural standing. Through this FES controller, FES was applied bilaterally to the ankle plantarflexors and dorsiflexors of the participants while they performed the VFBT exercises. The range of stimulation intensity applied during FES+VFBT fluctuated between the minimal contraction threshold and 80% of the maximal tolerable threshold for each participant. These thresholds were determined at the beginning of each training session by identifying the participant's minimal contraction threshold (i.e., the smallest stimulation current used to elicit a palpable minimal contraction) and the maximal tolerable threshold (i.e., the largest stimulation intensity that the participant was able to comfortably tolerate) for each muscle group in a sitting position, with current increasing by 2 mA. The stimulation frequency was set to 40 Hz and the pulse duration was set at 300 μs. During the training sessions, stimulation current was regulated via the COP position in a closed-loop manner (24, 25). The location of the participant's COP and the location of the desired target was sent to the computer and fed back to the two FES (Compex Motion II, Compex Motion, Switzerland) devices (38), one for each leg, to apply the amount of current needed to assist the participant in completing the task (see Figure 2A).

### Acceptability of Intervention

A series of semi-structured interviews were conducted with each participant immediately post-training and at 8 weeks post-training (see Figure 1). Using a semi-structured interview guide (see Table 2), participants were asked about their experience regarding the FES+VFBT intervention by a researcher (JU or KEM) who was not directly involved in the delivery of the training. Specifically, they were asked about what aspects of the training program they liked or disliked and what they found challenging. The interviews were audio-recorded and transcribed verbatim following the interview. Following transcription, each interview was analyzed using conventional content analysis (39) to code items and develop categories to identify themes. Two reviewers (DJH and JU) independently read each transcript multiple times and highlighted quotes. These quotes were then condensed, while preserving the core meaning (40), and assigned a code. Categories were formed by grouping together related codes and then interpreted to generate themes to address their underlying meaning.

**Table 2:** Semi-structured interview guide.

**We would like to hear about your experiences with FES for standing balance.** 1) What went well? 2) What was challenging? 3) Would you recommend balance training to another individual with an incomplete spinal cord injury? What advice would you give to someone who was about to begin the training program? 4) What did you like most about program? What did you dislike? 5) How do you think the program could be improved? Do you have suggestions for things that we could do differently?

*FES, functional electrical stimulation*.

## Results

Participant demographic and injury-related variables (Table 1), as well as their baseline scores on the clinical (Table 3) and biomechanical measures (Table 4), are provided. Participants were between 55 and 68 years of age, four out of the five participants were female, and four out of five participants had non-traumatic iSCI. Average baseline BBS and mini-BESTest scores ranged from 24.3 to 45.3/56 and 5.3 to 15.3/28, respectively. All participants completed 12 FES+VFBT sessions and no training-related adverse events were reported. No biomechanical assessment data were reported for Participant 2 due to her inability to consistently complete the two tests at all assessments due to fatigue.

**Table 3:** Mean (Standard Deviation) of baseline clinical performance and score at follow-up assessments.

**Participant**					
**BBS (/56)**	**1**	**2**	**3**	**4**	**5**
Baseline	24.3 (1.53)	28.3 (4.62)	38.0 (3.46)	45.3 (1.53)	27.3 (3.06)
Post-training	27	31	42	48	30
4 weeks post-training	30^*^^∧^	33^∧^	45^*^^∧^	45	38^*^^∧^
8 weeks post-training	28^*^	32	42	45	32
**Mini-BESTest (/28)**	**1**	**2**	**3**	**4**	**5**
Baseline	5.3 (0.577)	11.3 (1.15)	11.3 (2.31)	15.3 (0.577)	6.0 (0.0)
Post-training	9^*^	17^*^^∧^	12	12	7^*^
4 weeks post-training	10^*^^∧^	12	9	15	8^*^
8 weeks post-training	5	13	9	13	7^*^
**ABC Scale (%)**	**1**	**2**	**3**	**4**	**5**
Baseline	66.25 (0.625)	39.79 (4.02)	73.33 (7.91)	62.71 (1.91)	39.38 (1.65)
Post-training	60.63	31.88	78.13	63.13	46.25^*^
4 weeks post-training	70.63^*^	41.88	81.88	60.63	41.88
8 weeks post-training	66.88	39.38	85.63	63.13	46.88^*^

*BBS, Berg Balance Scale; Mini-BESTest, Mini-Balance Evaluation Systems Tests; ABC, Activities-specific Balance Confidence Scale*.

* an improvement >2 standard deviations compared to the mean baseline value

∧ * an improvement greater than or equal to minimal detectable change (MDC). Values in bold indicate improvements that are both >2 standard deviations compared to the mean baseline value and greater than or equal to the MDC*.

**Table 4:** Mean (Standard Deviation) COP parameters during eyes open quiet stance.

**Participant**				
**AP mean COP velocity (mm/s)**	**1**	**3**	**4**	**5**
Baseline	46.35 (5.03)	16.75 (4.56)	17.07 (3.49)	17.27 (4.18)
Post-training	27.96^*^ (2.91)	16.14 (1.34)	14.73 (0.798)	16.01 (3.51)
4 weeks post-training	25.92^*^ (5.49)	13.74 (1.83)	20.23 (3.41)	16.90 (1.29)
8 weeks post-training	31.64^*^ (6.36)	9.57 (1.39)	18.08 (2.81)	19.31 (1.04)
**ML mean COP velocity (mm/s)**	**1**	**3**	**4**	**5**
Baseline	37.93 (3.45)	14.52 (3.02)	10.54 (2.25)	8.90 (1.25)
Post-training	32.23 (3.69)	17.16 (4.42)	12.69 (5.24)	9.14 (1.43)
4 weeks post-training	31.16 (2.00)	10.73 (2.84)	18.16 (6.15)	8.88 (0.494)
8 weeks post-training	34.81 (7.61)	10.66 (2.67)	16.13 (3.21)	1.04 (0.984)
**AP COP RMS displacement (mm)**	**1**	**3**	**4**	**5**
Baseline	12.29 (1.73)	6.27 (1.75)	6.37 (0.851)	8.16 (1.48)
Post-training	12.32 (2.04)	5.90 (0.415)	7.57 (0.875)	5.72 (1.10)
4 weeks post-training	9.88 (1.11)	5.01 (0.761)	8.71 (1.14)	6.81 (1.10)
8 weeks post-training	13.94 (5.39)	4.28 (1.27)	8.10 (0.767)	8.81 (1.74)
**ML COP RMS displacement (mm)**	**1**	**3**	**4**	**5**
Baseline	11.62 (2.67)	7.67 (0.878)	3.59 (1.46)	5.22 (0.937)
Post-training	9.28 (1.45)	10.48 (1.04)	8.79 (5.14)	4.54 (0.825)
4 weeks post-training	8.75 (0.547)	4.88^*^ (1.42)	9.71 (3.46)	4.43 (1.22)
8 weeks post-training	11.07 (1.87)	5.38^*^ (1.30)	8.24 (1.56)	4.95 (0.227)

*AP, Anterior-posterior; ML, Medial-lateral; COP, Centre-of-pressure; VEL, Mean velocity; RMS, Root-mean-square*.

* *an improvement >2 standard deviations compared to the mean baseline value*.

### Clinical Assessment

Following the completion of FES+VFBT, improvements from baseline scores that exceeded 2SD (see Table 3) were observed on the BBS at 4 weeks post-training (Participant 1, 3, and 5) and 8 weeks post-training (Participant 1). Improvements >2 SD were observed on the mini-BESTest (see Table 2) immediately post-training (Participant 1, 2, and 5), 4 weeks post-training (Participant 1 and 5), and 8 weeks post-training (Participant 5). Improvements >2 SD were observed on the ABC Scale (see Table 2) immediately post-training (Participant 5), 4 weeks post-training (Participant 1), and 8 weeks post-training (Participant 5). Only two individuals (Participant 1 and 5) demonstrated improvements >2 SD on each of the three clinical assessments for at least one time point.

Using the MDC reported above for the BBS, clinically relevant changes were observed at 4 weeks post-training (Participant 1, 2, 3, and 5) and at 8 weeks post-training (Participant 5). Using the MDC reported above for the mini-BESTest, clinically relevant changes were observed immediately post-training (Participant 2) and at 4 weeks post-training (Participant 1). Using the MDC reported above for the ABC Scale, no clinically relevant changes were observed (see Table 3).

### Biomechanical Assessment

**Figure 3:**
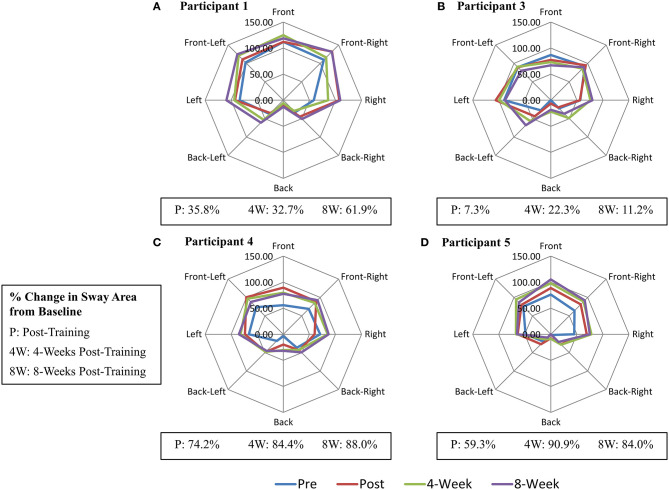
Change in Maximal Centre-of-Pressure (COP) Excursion (mm) during Limits of Stability Test. **(A)** Participant 1. **(B)** Participant 3. **(C)** Participant 4. **(D)** Participant 5.

Following the completion of FES+VFBT, a decrease in mean COP velocity >2 SD was seen in the AP direction for one participant (Participant 1) immediately post-training and at 4 and 8 weeks post-training. A decrease in COP RMS displacement was seen in the ML direction for one participant (Participant 3) at 4 and 8 weeks post-training (see Table 4). Following the completion of FES+VFBT, an increase in COP excursion during the limits of stability test (see Figure 3) was observed in all participants immediately post-training, with improvements ranging 7.3–74.2% greater than baseline values. Improvements were either maintained or further increased relative to baseline values at 4 and 8 weeks post-training (see Figure 3). Values for maximal COP excursion in the backwards direction are missing at the baseline and 8 weeks post-training assessments for Participant 5 and at the baseline assessment for Participant 3 due to technical errors.

### Acceptability of Intervention

Following the coding of the individual transcripts, five categories were identified: (i) the role of VFBT, (ii) the role of FES, (iii) the role of the research team, (iv) the role of the harness, and (v) scheduling and commitment to FES+VFBT. Together, these five categories formed the following theme: numerous factors impacting training performance.

Aspects of the training program that were perceived as being beneficial included the repetition and routine of the VFBT standing exercises, the addition of the stimulation, the support from the research team and the safety harness. Participants felt that the training program provided a safe environment to practice the VFBT exercises and other challenging activities. The presence of the safety harness and the research team allowed them to focus their attention on performing the movements that the exercises promoted without having to be concerned with falling. Participants also noted that participation in the program required commitment as not only was the program physically challenging, but traveling to the training sessions was challenging for some participants as well. Despite the required commitment, all participants indicated that they would have liked the program to last longer than 12 sessions.

#### The Role of VFBT

Participants reported a number of different factors related to the nature of VFBT that they felt contributed to the success of the program; namely movement, repetition, challenge and feedback. Participants commented that they enjoyed the activity and movement offered by the intervention and that the training taught them how to stand properly. Participant 1 explained how he believes that “*any movement is good movement [and] any activity is good activity”* while Participant 3 mentioned that she liked learning “*how to put [her legs] in certain positions that help to actually stand better.”* They also appreciated how the training made them work and use their muscles as well as the routine that the training sessions provided. Participant 4 found that completing the exercises required “*using the muscles, the right leg, the left leg*” which she enjoyed. Participant 2 said she enjoyed the program because of “*the routine and the habit that [she gets] into coming”* and “*because it makes [her] work.”*

The repetition offered by the training exercises led to increased feelings of confidence and some individuals mentioned how they looked forward to the training because it made them feel independent as they stood and performed the exercises. Participant 2 believed that “*practice makes perfect”* and felt her confidence was positively affected by the repetition of the training exercises. Participant 4 explained how she looked forward to standing in front of the computer screen during her training sessions because “*[she] felt very independent, [she] felt stronger, that [she] can do it while standing.”*

Many of the participants found the exercises to be challenging, but enjoyable. Each exercise required the participants to shift their COP (represented on screen as a red marker) and maintain their position within a presented target. In some exercises, the location of the next target was unknown, which made it difficult to plan movements and shift the COP. Participant 4 perceived controlling the marker, and moving it toward the presented target during the exercises, as a “*good, high level of challenge.”*

Participant 3 found the standing itself to be quite challenging because it had been some time since she had been in that position and it felt as if she had forgotten how to stand properly. She explained how trying “*to stay standing still, even for a couple of seconds, seems like a lot to [her]”* and found that the most challenging part was trying to “*stay inside the center [of the targets].”*

In contrast, Participant 1 felt that by the end of the training he was quite comfortable with several of the exercises and wanted something more challenging as he felt he had “*mastered a lot of it.”* Due to the visual nature of the exercises, participants could see how they were controlling their COP as they moved toward the targets. Participant 1 felt that when he performed the exercises that his movement was “*a very smooth shift back-to-back; smooth and controlled.”* Participant 3 found that the exercises highlighted her strengths “*a little better when the [marker] moves from in the left side”* and weaknesses when moving in certain directions and recognized that the exercises were designed to encourage her to move in a variety of different ways.

Participants were also able to see in real-time how they were performing on the exercises based on how much time they had spent in the targets or how many targets they had managed to move toward. Participant 1 commented that the results could provide additional motivation for some participants as they attempt to improve their performance since “*everybody's sort of results-oriented”*, but explained that “*you come into it with motivation anyways”* and how he didn't base his effort on his performance.

#### The Role of FES

The use of FES in the training was well-received by the participants. Participant 1 believed that applying the FES had “*reawakened those muscles”* and emphasized “*it's a good feeling.”* For Participant 4, she described that with the stimulation it felt “*like the muscles were working.”* Participant 5 believed that the use of FES is the reason “*why [she] got more confident”* because she hadn't “*[changed] anything other than that”* and wasn't doing any additional exercise aside from the intervention.

Participant 3 commented that at times the FES could feel quite strong when she would “*start standing and start playing the games”*, but that usually she was too focused on the exercises to really pay attention. During standing, and while performing the exercises, participants explained they were aware of the stimulation and how it assisted them. Specifically, they felt that the FES helped as they stood and moved in different directions toward the targets and especially noticed their muscles being engaged as they pushed past their typical range of stability or if they felt like they were about to fall. Participant 4 remarked that “*the stimulation helped [her] a lot; helped her standing, moving right, moving left, forwards, but not…backwards.”* For Participant 1, he explained that he was aware of the FES activating his muscles during the games and “*especially if [he] was going out somewhere…out of [his] range…like really trying to hit this [target]…[he] felt them; they were on.”* Likewise, Participant 3 noticed that “*when [she was] almost falling, [she] might feel a little bit more there, the stimulation…”* would kick in and contract the muscles.

#### The Role of the Research Team

Participants found that the encouragement and support offered by the members of the research team had a positive impact on their training experience: “*The people. That's what makes everything, right? I mean they were really, really a great group of people. They were keen, and they were knowledgeable, and they were enthusiastic, and they were supportive, and they were encouraging…you can't beat those attitudes”* (Participant 2).

Over the course of the intervention, setting up the training sessions became easier and more efficient, which was beneficial for both the participants and the research team. The presence of the research team and the lab environment also instilled confidence in the participants, as they were able to safely practice activities that would otherwise be unable to attempt in their own homes without the fear of worsening their situations. As Participant 5 explained: “*they are here, the harness is here, I can practice…but at home, no, I can't do that; I don't want to get worse.”*

#### The Role of the Harness

Throughout the training sessions, participants were securely fastened in a safety harness to protect against a fall as they performed the four FES+VFBT exercises. The majority of the participants found the harness beneficial because it made them feel secure and encouraged them to practice the exercises without worrying: “*For me, without the harness, trying to do those things, you have to focus on whatever exercise you're doing; it takes a lot…I'm aware of the sling, I know it's there. I know that I don't have to be apprehensive; I can try and go to the end of my range. If I miss, so what? The sling is there. I think if I didn't have the sling and I was freestanding I'd be pretty apprehensive about doing some of those things… I think it encourages [you] because you know you're safe… I'm worried, about safety all the time…because you know, if I go down it's hard to get back up again.”* (Participant 1) However, Participant 3 suggested that “*sometimes [she didn't] want to wear the harness”* in order to see what she was capable of doing without any support.

#### Scheduling and Commitment to FES+VFBT

Participants found the FES+VFBT training schedule to be manageable, but explained that they would have preferred the program to last for longer than 12 training sessions as they found the intervention to be quite short. Three training sessions were completed per week for a total of 4 weeks. Participants appreciated the day off in between each session as it allowed them time to recover between sessions and allowed them to have a life outside of the intervention. Over the course of the training, Participant 2 felt that she “*was able to improve a little bit…play the games better”* but explained that it wasn't always consistent. She admitted “*there were times where it was very tiring and [she] just couldn't get [her] body to do what [she] wanted it to do”* which could be frustrating.

Participant 3 appreciated the routine offered by the intervention as it encouraged her “*to get up from the bed…get dressed and go out.”* Although the training sessions did require a lot of energy, she did “*feel like it [was] helping [her] somewhat”* and believed that it was important to try.

For others, commuting to the training session was the most tiring aspect. Participant 5 explained how she would like “*to do more activity, but the traveling is killing [her]”* After waiting for her WheelTrans ride and then sitting in the vehicle, she is exhausted from her travels and doesn't “*want to do exercise”* as a result.

## Discussion

The outcomes of a 4 week FES+VFBT program on the balance ability and balance confidence of five individuals with chronic motor iSCI are described here. This case series provides quantitative evidence that FES+VFBT can impact static and dynamic balance performance as assessed using clinical and biomechanical measures of balance.

Improvements that exceeded 2 SD were seen on the BBS and/or the mini-BESTest in four of the five participants following training, while only two participants exhibited increases >2 SD on the ABC scale. Overall, fewer participants achieved a magnitude of improvements on the clinical measures reported to be clinically relevant (i.e., the MDC), in particular on the mini-BESTest and the ABC Scale (see Table 3). The methods used to calculate the MDC for these two clinical measures was based on the statistical distribution of scores amongst a sample of individuals with motor iSCI (30, 34). Hence, the patient's perspective is lacking in these values representing clinically relevant change (41), and this should be considered when interpreting the quantitative results. Reports of increased confidence by several participants suggest that they experienced meaningful changes as a result of FES+VFBT, despite the lack of quantitative evidence, and highlight the importance of including qualitative components when evaluating interventions.

While improvements in standing balance ability and balance confidence were observed following training, only two participants maintained the improvements (i.e., >2 SD) at 8 weeks post-training. It is possible that the intensity (1 h, 3 days/week) or the duration (4 weeks) of the training program was insufficient to produce long-lasting effects. In another study involving the iSCI population, Tamburella et al. (13) demonstrated significant improvements in all balance parameters 2 months following the completion of an 8 week training program that involved a total of 40 h of training (40 sessions; 5 times/week; 40 min of gait training; 20 min of visual biofeedback balance training). Increasing the total dosage of FES+VFBT could be pursued in further research. The fact that only about 20 min of the 1 h FES+VFBT session was being spent on the actual therapy necessitates increased efficiency in the delivery of the FES+VFBT intervention. One way to improve the efficiency of FES+VFBT would be to reduce the amount of time spent calibrating the electrical stimulation parameters. Despite this small dosage we did manage to observe improvement on clinical and biomechanical assessments of balance, which suggests that VFBT is a promising intervention for people living with motor iSCI.

FES+VFBT had a limited effect on balance confidence, at least according to the ABC Scale. Many of the tasks queried on the ABC Scale (35) are ambulatory tasks (e.g., “How confident are you that you will not lose your balance or become unsteady when you walk outside the house to a car parked in the driveway?”). As FES+VFBT focused only on standing tasks, it is not surprising that the intervention had minimal impact on ABC Scale scores. Motor skill learning is known to be task-specific, for example, spinalized cats who practiced standing on all four limbs did not improve their ability to step, and vice versa (42). Hence, repetitive practice of standing may lead to improvements in standing ability and confidence, but may not improve the ability to perform more dynamic tasks, such as walking. The focus on standing balance was an appropriate choice of intervention for the participants of this study as none were able to ambulate without a gait aid and/or physical assistance at study outset.

All five individuals showed improvements in the maximal COP excursion area following FES+VFBT that ranged from 7.3 to 90.9% times greater than initial baseline performance (see Figure 3). Since three out of four VFBT exercise encouraged the participants to shift their COP in a similar manner to the limits of stability test, it is possible that there was some transfer in motor skill from training to this task. In contrast, few participants showed improvements in measures of postural sway during quiet standing after FES+VFBT. Again, this finding may reflect the task-specific nature of motor learning. Only one of the four VFBT exercises involved standing still; hence participants spent more time practicing dynamic balance tasks than static balance tasks. However, Sayenko et al. (12) included similar visual feedback training exercises in their study and found significant decreases in postural stability measures during eyes open quiet stance following training completion, with the exception of mean COP velocity in the ML direction. Participants in this previous study performed six COP-based games in total, with only one game involving quiet standing; in this case, practice of dynamic balance tasks did result in improvements in the static balance task. The improvements in postural sway measures observed during quiet stance, in the study by Sayenko et al. (12), may be attributed to a greater dosage of training. In their study, participant also completed three 1 h training sessions per week, for a total of 12 sessions; however, due to the simpler experimental set-up in the study (i.e., no FES), it is possible that more time could have been allocated to VFBT during the 1 h training sessions. Differences in the level of standing ability between the current study and the study by Sayenko et al. (12) might also explain the differing results. In the study by Sayenko et al. (12) participants were able to stand for at least 5 min without an assistive device, which was a greater level of standing tolerance than the participants in the current study.

All five participants adhered to the training schedule, supporting the feasibility of the FES+VFBT intervention for those who enrolled in the study. Moreover, through qualitative inquiry, participants highlighted several specific components of the program that worked well, including the repetition of the VFBT exercises and the challenges associated with the unpredictability of the target locations. The ability to practice these movements in a safety harness and under the supervision of the research team enabled participants to focus on performing the exercises in a safe and controlled manner without worrying about a fall. Participants emphasized that three sessions per week was appropriate, but expressed their desire to extend the duration of the program a few more weeks (i.e., 18–24 total training sessions). Recruitment and enrolment into the FES+VFBT program, however, proved more challenging. Fifteen individuals were recruited via flyers, but only five participants were included in our sample. Five individuals did not respond following initial contact, four individuals did not meet the inclusion criteria, and one individual declined to participate due to concerns regarding time commitment. A 2:1 screening to recruitment ratio for SCI rehabilitation interventions (43) has been reported, which may explain our small sample. We reported a 3:1 screening to recruitment ratio for this study. The use of FES in this study may have contributed to this increased ratio. While the application of surface FES is not an invasive intervention, it may be viewed as more intrusive than other exercise-based interventions. Participation in FES also requires clearance of numerous contraindications and precautions (44) that may result in a higher number of screens fails. Our inclusion criteria also excluded individuals with balance abilities >46 on the BBS and individuals with a neurological level of injury below T12.

## Limitations

There are a few limitations of the study to acknowledge. First, the characteristics of the studied sample do not reflect the characteristics of the larger SCI population in Canada. Within the studied sample, 80% of participants were female, and 80% had a non-traumatic SCI. The characteristics of this sample do not reflect the prevalence of SCI among the Canadian population. Of the individuals with an SCI living in Canada, 26% are female (45). The increased representation of female participants within our sample may be explained by a greater fear of falling reported by women in comparison to men (46). As a result, this may increase their willingness to participate in balance training activities. However, due to our limited sample, it is not possible to say whether sex and gender influence participation in balance interventions, but it would be beneficial if future, larger studies considered sex and gender influences.

In our current study, four participants had non-traumatic SCI. This mixture of SCI etiologies (i.e., traumatic and non-traumatic) is a result of our sample of convenience. However, our objective was to develop an intervention that is appropriate and applicable for all individuals with SCI. Since the etiology of SCI is heterogeneous, with approximately equal prevalence of traumatic and non-traumatic etiologies of SCI in Canada (47) we believe that our sample reflects this reality. However, future work should involve larger randomized control trials in order to evaluate the efficacy of FES+VFBT in comparison to conventional balance training among individuals and could include sub-groups of SCI.

The experimental design of the study could also be considered a limitation. However, given the early stages of the development of FES+VFBT, we believe it to be appropriate. When developing and evaluating novel rehabilitation interventions, uncontrolled trials focused on evaluating feasibility and appropriateness often precede larger randomized controlled trials (27, 28).

While this current study does not include a typical control group, by adopting the single-subject experimental design, each participant serves as their own controls (26, 28). Having the baseline period as long as the intervention period allows for a comparison between the novel intervention and the “usual care” during the chronic phase of SCI. However, as this is a case series, it is not possible to draw conclusions on the efficacy of the FES+VFBT intervention for standing balance among individuals with SCI.

## Conclusions

Improvements were seen in four of five participants on at least one of the clinical balance scales following training, with less impact on balance confidence as measured by the ABC Scale. The area of maximal COP excursion increased for all participants, while there was little effect on quiet stance assessments. All five participants appreciated the opportunity to practice challenging standing balance tasks in a safe environment with the assistance of FES and would have preferred a longer intervention period. While the majority of participants did not sustain their improvements at 8 weeks post-training, the fact that FES+VFBT was able to elicit improvements in balance ability despite a small training dosage suggests that it is a promising intervention for standing balance rehabilitation among individuals with iSCI.

## Data Availability Statement

The datasets generated for this study are available on request to the corresponding author.

## Ethics Statement

The studies involving human participants were reviewed and approved by University Health Network and University of Toronto Research Ethics Boards. The patients/participants provided their written informed consent to participate in this study.

## Author Contributions

KEM and KM conceived and designed the study. DH and JL collected and analyzed the data. JU and KEM conducted the interviews. DH and KEM interpreted the results. DH drafted the manuscript. KEM, JL, JU, and KM critically revised the manuscript. All authors approved the final version of the manuscript.

## Conflict of Interest

The authors declare that the research was conducted in the absence of any commercial or financial relationships that could be construed as a potential conflict of interest.
